# Estimating the incidence of enteric fever in children in India: a multi-site, active fever surveillance of pediatric cohorts

**DOI:** 10.1186/s12889-018-5498-2

**Published:** 2018-05-03

**Authors:** Jacob John, Ashish Bavdekar, Temsunaro Rongsen-Chandola, Shanta Dutta, NSSEFI Collaborators, Gagandeep Kang, Jacob John, S. Arun Karthikeyan, VenkataRaghava Mohan, Veeraraghavan Balaji, Manikandan Srinivasan, Sindu Kulandaipandian Natarajan, Ira Praharaj, Winsley Rose, Ashish Bavdekar, Ankita Shrivastava, Sonali Sanghavi, Sanjay Juvekar, Temsunaro Rongsen-Chandola, Bireshwar Sinha, Deepak More, Alok Arya, Nidhi Goyal, Chandra Mohan Kumar, Aparna Chakravarty, Shanta Dutta, Suman Kanungo, Byomkesh Manna, Pranab Chatterjee, Gagandeep Kang

**Affiliations:** 1 Christian Medical College, Vellore, India; 2 KEM Hospital and Research Centre, Pune, India; 3 Society for Applied Studies, New Delhi, India; 4 National Institute for Cholera and Enteric Diseases, Kolkata, India

**Keywords:** Cohort study, Incidence, Typhoid fever, Salmonella Typhi, Community surveillance, Active surveillance

## Abstract

**Background:**

Salmonella Typhi is responsible for about 20 million episodes of illness and over 140,000 deaths annually globally. South Asia has the highest documented burden of typhoid and is home to the multi-drug resistant H58 strain that makes treatment more challenging. The WHO recommends the use of Typhoid Conjugate Vaccines in typhoid endemic countries. Decisions on the preferred immunization strategy should be based on an analysis of disease burden, availability, affordability, and operational feasibility. Typhoid vaccines have so far remained unimplemented as public health measures because of a perceived decline in typhoid burden in recent years. The apparent decline, based on hospital reports, may be a result of rampant antimicrobial use in the community and therefore estimation of disease incidence at the community is necessary to better measure disease incidence and transmission.

**Methods:**

Age-specific incidence of typhoid fever in children between 6 months and 15 years will be estimated in four community based cohorts in varied settings across India using standardized protocols for active fever surveillance in the community. Data will be collected on secured cloud infrastructure using a combination of android and web-based real-time data collection tools. Blood cultures will be done for children with fever lasting 3 or more consecutive days using automated blood culture systems. Those with blood-culture confirmed typhoid fever will be followed up till 90 days to estimate costs and clinical outcomes of the illness episodes. Environmental factors, access to safe water, sanitation, hygiene, food hygiene, demography, population density and socioeconomic status will be assessed periodically to characterise risk factors and permit extrapolation of burden to similar risk settings.

**Discussion:**

With the availability of licensed typhoid conjugated vaccines in India, it is important to consider whether the burden of disease is present and sufficient to require the use of vaccine in addition to other interventions. Active case finding in the community permits the detection of cases that would be missed in facility-based surveillance systems. Understanding the age distribution, burden, cost-of-illness and transmission of disease is essential to plan interventions and predict their potential impact.

**Trial registration:**

The surveillance has been prospectively registered in the Clinical Trial Registry of India (CTRI/2017/09/009719) on 12 September 2017.

## Background

Salmonella Typhi is the leading cause of enteric fever cases responsible for between 12 and 21 million illness episodes and over 140,000 deaths each year globally [1, 2]. The disease is largely prevalent in the low and middle income countries of South Asia. Three community-based cohort studies conducted between 1995 and 2006 in India estimated the incidence of culture confirmed typhoid at 377 (178–801) per 100,000 person years with the highest incidence in early childhood [3–5]. The burden of enteric fever is predicted to worsen with the emergence of multi-drug resistant strain H58 in this region.

The World Health Organization (WHO) in its position paper on typhoid published in 2008 [6] recommends the use of typhoid vaccines to prevent infection in high-risk populations, particularly where antibiotic resistance is prevalent. These recommendations have remained unimplemented in India partly due to a perceived decline in the incidence and severity of typhoid fever in recent hospital-based reports accompanied with evidence of heterogeneous distribution of disease. These findings of declining disease burden in hospital based studies can partly be attributed to the indiscriminate early use of antibiotics to treat febrile illness. Newer antibiotics, that are increasingly accessible over the counter, have been shown to decrease transmission of this host-restricted organism when administered appropriately, early in disease [7–9]. Furthermore, antibiotics decrease culture confirmation by achieving defervescence in ambulatory care where cultures are generally not performed and by decreasing the sensitivity of blood culture post antibiotic therapy. In the absence of an alternative diagnostic assay of sufficient diagnostic accuracy, estimation of disease burden continues to rely on isolation of the organism by blood culture that is likely to be influenced by prior treatment and healthcare seeking behaviour.

While it is challenging to disaggregate the complex interplay between antimicrobial use, antimicrobial susceptibility and disease burden, estimating age-specific incidence of typhoid in the community by investigating febrile episodes prior to antimicrobial therapy will provide a better understanding of current disease burden and its transmission dynamics.

Estimating the burden of disease in children who are the most likely beneficiaries of typhoid conjugate vaccines in national immunization programs will permit us to predict the potential utility of such a vaccine introduction. In this study we will estimate the risk of developing typhoid in cohorts of children aged between 6 months and 15 years through active weekly surveillance for suspected typhoid fever. This approach will permit us to identify febrile illness episodes, document antimicrobial use and ensure blood culture prior to antimicrobial therapy in those with suspected typhoid fever.

## Methods

### Hypothesis

Active, community-based surveillance will estimate the true incidence of typhoid fever and provide a better understanding of transmission of typhoid in the population than available data from hospital based extrapolations.

#### Objectives

The two-fold objectives of this multi-site active surveillance are as follows

1. To estimate the age specific incidence of culture confirmed typhoid fever in 6 months-4 years, 5–9 and 10–14 years age groups across four geographically distinct sites in India
2. To describe the incidence of acute febrile illness and its associated treatment practices in the community

#### Inclusion and exclusion criteria

##### Inclusion criteria

- A child between 6 months and 13 years + 364 days of age.
- A child who is likely to remain in the study area for the duration of the study.

##### Exclusion criteria

- Non-consent

### Study design

We will recruit 6000 eligible children from each of the four geographically distinct study sites across India. Children are eligible to participate if they are between 6 months and 13 years + 364 days of age, likely to be in the study area for the duration of the study and are willing to receive investigations in the event of febrile illness. All eligible children living in the designated study area will be offered participation and the enrolment will continue till 6000 children have been recruited into each site’s cohort. If multiple eligible children are present within a household, all of them will be eligible to participate. Written informed consent will be obtained from the primary caregiver in addition to assent from age-eligible children.

Active weekly fever surveillance will be performed by trained study personnel either through home visits or by telephonic contacts through the course of study participation. Each participant will be followed for 24 months or until the child attains 15 years of age, whichever is earlier. The study team will ensure at least one face to face contact with the child and the primary care giver every 4 weeks. During these weekly contacts, details of any illness, visits to a health facility and treatment received since the previous visit will be recorded for each participant within the household. If contact with the parents/primary caregiver is not established for two consecutive weeks, then a supervisory visit will be made to confirm that the child is unavailable for surveillance. Parents are encouraged to report any fever episode to the study team at the earliest in addition to reporting them at weekly visits by study personnel.

In the event of fever, a daily follow up will be established until there are three consecutive fever free days. A digital thermometer and a fever diary card will be provided, and primary caregivers will be trained to use the thermometer and document the temperature three times a day. The study team will also document the temperature of the child in the fever diary card during home visits. At each daily contact during the fever follow up, the study team will document information pertaining to the previous calendar day including the highest documented temperature. If the child has any serious illness the parents will be encouraged to seek healthcare from their preferred provider. The family will be informed the need to avoid antibiotics and over the counter medicines that are not prescribed by their physician.

Any reported fever for 3 or more consecutive days will be designated as ‘Suspected Typhoid Fever’ and the child will be encouraged to visit the study clinic. At the study clinic the child will be evaluated by a study physician on the fourth day of fever and if there is history of fever in the preceding 12 h a blood culture will be requested. If the child has been afebrile for over 12 h, unless clinically indicated the blood culture will be deferred. Any subsequent fever within the next 24 h will warrant a blood culture. Prior antimicrobial therapy will not be a contraindication for blood culture. If a child has received a blood culture in the previous 14 days, blood culture will be withheld unless it is requested for by the clinician treating the participant.

### Laboratory methods

A dedicated laboratory team will be present at each site for collection of blood samples and subsequent processing using automated blood culture systems using standard operating procedures across sites. For children between 6 months and 1 year, 3 mL will be inoculated into a paediatric blood culture bottle. For children above 1 year of age, 5 mL of blood will be inoculated into a paediatric blood culture bottle. Additional blood may be drawn for investigations requested by the treating physician. The total volume of blood drawn must not exceed 2 mL per kg body weight. Blood samples will be transferred to the site laboratory facility within 4 h of sample collection at ambient temperature. Where blood cultures do not provide a definite diagnosis and fever persists for 7 or more days, additional serology based tests may be performed as clinically indicated but these will not contribute to the estimates of typhoid incidence. *S.* Typhi isolated from other normally sterile sites will also contribute to confirmed typhoid fever estimates. All typhoid isolates will undergo antimicrobial susceptibility testing and will be archived for future genomic characterisation. All other significant culture isolates including *S. Paratyphi* will be documented.

### Sample size

The incidence of acute typhoid fever in children aged 6 months to 15 years in India was previously estimated to be between 6 and 12 per 1000 child years (CY) with the highest rates in 1–4 year olds [4, 10]. If community-level incidence has declined as hospital-based estimate suggest, the current incidence in this age-group would be between 1 and 5 per 1000 CY. Recruiting, at each site, a cohort of 5600 children between 6 months and 13 years + 364 days will provide 8400 child years of observation over 18 months that would be distributed approximately as 2400 child years in the 6-month to 4 years, and 3000 child years in the 5–10 and 11–14 age groups in an open cohort and assuming a uniform death rate. Accumulating 8400 child-years will exclude an incidence rate of 2/1000 with 95% confidence if there are 26 or more cases (IR > 3/1000) in the cohort. In turn, this sample size will be adequate to exclude at 95% confidence limits, a rate of 2/1000 in each of the 6 months-4 years, 5–10 and 11–14 years age groups in which the observed incidence is 4/1000 or greater using a Fishers exact test based on the formula (Armitage,1971; Snedecor & Cochran,1965) as described in Epidemiologic Analysis with a Programmable Calculator, 1979.

### Data management

The data for this multi-site surveillance will be centrally managed in a cloud based infrastructure that is appropriately secured to ensure participant confidentiality and compliance with local laws. In the community data will be gathered on android tablets using android application package *‘EntericFev’* developed in house and stored in PostgreSQL databases on an Amazon cloud EC2 instance. Clinic and laboratories will use a secured web browser based application to upload and edit data. A comprehensive audit trail enabled database management solution for data managers and a real-time dashboard for the project managers permit careful monitoring and implementation of the surveillance.

### Data analysis

Incidence rates will be calculated as the number of discrete events of illness (for confirmed typhoid fever and acute febrile illness) over the total person-time under observation and at risk. Recurrent events will be allowed to include multiple episodes of illness over the study period. The person-time for each child will begin from the date of enrolment to the end of follow-up or date of loss to follow-up. Time with illness and when children not under surveillance will be excluded from the calculation of person-time. Rates will be expressed as per 1000 child-years. The two-year study period will be divided into three age intervals (6 m – 4 years, 5-9 year and 10-14 years) determined by the chronological age. The incidence rates for each age interval will be calculated based on the number of children at risk in the beginning of each interval, the number who had the event and the number who were lost to follow-up. The age-specific rates will be calculated as the number of new cases of illness in the specific age interval divided by total number of person-years of follow-up contributed by all children at risk in this interval. Children will be allowed to move higher up in the age intervals. Because recurrent events of illness will be considered, we will account for correlation at the level of individual child using mixed effects poisson regression model. All statistical analysis will be performed using Stata 14.0 (Stata Corp).

### Risk characterisation of the population

Annual assessment of access to safe water, sanitation and hygiene, population density, demography and socioeconomic status will be performed for risk characterisation of the population.

### Typhoid sub-cohort

All children with culture-confirmed typhoid will be invited to participate in a longitudinal follow-up to estimate costs and outcomes of the illness episode. They will be contacted on the 14th, 28th and 90th days since the onset of fever. Trained study personnel will administer questionnaires to estimate costs at the end of the illness episode, and at 14 days and at 28 days from fever onset. The clinical outcomes of the illness episode will be documented at 28 and 90 days. A single blood sample of 3 mL will be collected at the 28th day visit to assess immune responses to the infection.

### Characteristics of study population

#### Vellore

The cohort will be established within a population of 48,000 that is part of the Vellore Demographic Surveillance System (VDSS) that was established in 2002 in the contiguous semi-urban settlements of Chinallapuram, Kaspa, Ramnaickanpalayam and Vasanthapuram. Within the VDSS, Christian Medical College (CMC) provides access to ambulatory medical care to children through a community clinic. In addition, children from this area access secondary and tertiary care at the hospitals of CMC located in the vicinity. Other public and private service providers within the area are generally willing to work with the research team.

#### Pune

The study cohort will be established in four villages namely Shikrapur, Koregaon Bhima, Vadu Budruk and Perane from the Vadu HDSS area, which is an independent system initiated in 2002 to monitor health trends, disease and vital events in the population served by the Vadu Rural Health Program. It is a well-defined rural area, with more than 170,000 people residing in 22 villages. This area is located 30 km from the Pune city and receives seasonal rains and has a predominantly agrarian economy. Health facilities include one rural hospital in the non-government sector, one public rural hospital and several health centres in the public sector and more than 30 small general and maternity hospitals in the private sector. There is established rapport with public and private healthcare providers in Vadu area. Shirdi Saibaba Hospital situated in Vadu village is a 35 bed multi-disciplinary rural hospital provides secondary level medical care and will form the base for the study team.

#### Delhi

The surveillance will be established in geographically contiguous areas of Sangam Vihar, Dakshinpuri and Madangir in South Delhi. These typical urban resettlement neighbourhoods have an estimated population of 1000,000 and an anticipated annual birth cohort of 20,000. The median family income is INR 7000 month. Almost all homes have access to a mobile phone. Being located in the heart of the city the residents have easy access to public transportation to tertiary hospitals that provide access to medical care to children in this community through dedicated clinics. Additionally, government clinics and private practitioners charging nominal fee are present in the area. The study team have been working in the area for several years and have other population based studies ongoing in the area.

#### Kolkata

In Kolkata, the study will be conducted in Wards 58, 59 & 66 of Kolkata Municipal Corporation area. These wards are situated in the eastern part of Kolkata, around 5–8 km from the National Institute for Cholera and Enteric Diseases where the study will be coordinated from. The area mainly consists of urban slums, densely populated, with narrow streets and lanes, having shared piped water supply and toilet facilities. The study team will establish fever clinics within the study area and work in close coordination with practitioners in the area. The study team has conducted several clinical research projects in this region.

## Discussion

Efficacious, licensed conjugate typhoid vaccine are currently available in India [11, 12]. Uncertainty about disease burden and distribution fueled by hospital-based literature suggesting declining burden in recent years has hindered the adoption of typhoid vaccines as a public health intervention [13, 14]. Examining the incidence, age distribution and transmission dynamics of typhoid fever through active surveillance studies in geographically distinct sites will enable us to uncover disease that remains undetected in hospital-based reports because of varying health seeking characteristics and the rampant use of antibiotic early in febrile illness prior to seeking healthcare in facilitates that perform appropriate diagnostics. Persisting transmission of typhoid in the communities where indiscriminate antibiotic use is rampant may lead to the emergence of multi-drug resistant typhoid haplotypes that will be increasing challenging to treat [15, 16].

The age distribution of typhoid not only indicates the force of infection but also enables policy makers to make informed decisions on the choice and timing of vaccines interventions. Multi-site, multi-year examination of incidence of typhoid is needed to ascertain whether targeted control strategies are more appropriate than universal immunization.

Active surveillance systems using blood culture for identifying cause of fever face several challenges. In the absence of point of care diagnostics with acceptable diagnostic accuracy, this surveillance will perform automated blood cultures on children with suspected typhoid fever. This process requires the aseptic collection of 3 to 5 ml of blood in febrile episodes, preferably before initiation of antibiotics. We propose a blood culture in those with three or more days of reported fever as the prevalence of antibiotic therapy in those with over 3 days of fever is substantial. We use reported fever over documented temperature as temperature documentation for fever has been inconsistent in prior studies in these population and may result in several suspected typhoid fevers remaining ineligible for culture.

Active surveillance studies are resource intensive and challenging to perform. However, in the absence of alternative diagnostic methods that are uninfluenced by health utilization patterns and prior antibiotics, these remain the gold standard to ascertain the transmission and age-specific incidence of typhoid in the community. Antimicrobial resistance and challenges in ensuring safe water and sanitation in the near term necessitates the consideration of the available vaccine interventions that remain unused. We believe demonstrating unequivocally the burden of typhoid in the community will spur public health managers to consider vaccines in conjunction with other preventive strategies for control of both typhoid and antimicrobial resistance.

## Abbreviations

CMC: Christian Medical College Vellore
CY: Child years of observation
WHO: World Health Organisation

## References

[1] Crump JA, Luby SP, Mintz ED (2004). The global burden of typhoid fever. Bull World Health Organ.

[2] Buckle GC, Walker CL, Black RE (2012). Typhoid fever and paratyphoid fever: systematic review to estimate global morbidity and mortality for 2010. J Glob Health.

[3] Bahl R, Sinha A, Poulos C, Whittington D, Sazawal S, Kumar R (2004). Costs of illness due to typhoid fever in an Indian urban slum community: implications for vaccination policy. J Health Popul Nutr.

[4] Sur D, Ochiai RL, Bhattacharya SK, Ganguly NK, Ali M, Manna B (2009). A cluster-randomized effectiveness trial of vi typhoid vaccine in India. N Engl J Med.

[5] Ochiai RL, Acosta CJ, Danovaro-Holliday MC, Baiqing D, Bhattacharya SK, Agtini MD (2008). A study of typhoid fever in five Asian countries: disease burden and implications for controls. Bull World Health Organ.

[6] Typhoid vaccines: WHO position paper. Wkly Epidemiol Rec. 2008;83(6):49-59. PubMed PMID: 18260212. 18260212

[7] Chinh NT, Parry CM, Ly NT, Ha HD, Thong MX, Diep TS (2000). A randomized controlled comparison of azithromycin and ofloxacin for treatment of multidrug-resistant or nalidixic acid-resistant enteric fever. Antimicrob Agents Chemother.

[8] Parry CM, Hien TT, Dougan G, White NJ, Farrar JJ (2002). Typhoid fever. N Engl J Med.

[9] Parry CM, Ho VA, Phuong l T, Bay PV, Lanh MN, Tung l T (2007). Randomized controlled comparison of ofloxacin, azithromycin, and an ofloxacin-azithromycin combination for treatment of multidrug-resistant and nalidixic acid-resistant typhoid fever. Antimicrob Agents Chemother.

[10] Sinha A, Sazawal S, Kumar R, Sood S, Reddaiah VP, Singh B (1999). Typhoid fever in children aged less than 5 years. Lancet.

[11] Mohan VK, Varanasi V, Singh A, Pasetti MF, Levine MM, Venkatesan R (2015). Safety and immunogenicity of a vi polysaccharide-tetanus toxoid conjugate vaccine (Typbar-TCV) in healthy infants, children, and adults in typhoid endemic areas: a multicenter, 2-cohort, open-label, double-blind, randomized controlled phase 3 study. Clin Infect Dis.

[12] Jin C, Gibani MM, Moore M, Juel HB, Jones E, Meiring J (2017). Efficacy and immunogenicity of a Vi-tetanus toxoid conjugate vaccine in the prevention of typhoid fever using a controlled human infection model of Salmonella Typhi: a randomised controlled, phase 2b trial. Lancet.

[13] John J, Van Aart CJ, Grassly NC (2016). The burden of typhoid and paratyphoid in India: systematic review and meta-analysis. PLoS Negl Trop Dis.

[14] Mogasale V, Maskery B, Ochiai RL, Lee JS, Mogasale VV, Ramani E (2014). Burden of typhoid fever in low-income and middle-income countries: a systematic, literature-based update with risk-factor adjustment. Lancet Glob Health.

[15] Baker S, Duy PT, Nga TV, Dung TT, Phat VV, Chau TT (2013). Fitness benefits in fluoroquinolone-resistant Salmonella Typhi in the absence of antimicrobial pressure. elife.

[16] Divyashree S, Nabarro LE, Veeraraghavan B, Rupali P (2016). Enteric fever in India: current scenario and future directions. Tropical Med Int Health.

